# Energy and macronutrient intakes of Montenegrin adults: insights from the EFSA EU Menu National Survey (2017–2022)

**DOI:** 10.3389/fnut.2026.1741525

**Published:** 2026-03-05

**Authors:** Amil Orahovac, Snežana Barjaktarović Labović, Andrea Milačić, Aleksandra Martinović, Robert L. Mach

**Affiliations:** 1 Faculty of Food Technology, Food Safety and Ecology, University of Donja Gorica, Podgorica, Montenegro; 2 Center for Science, Department for Science, Projects, International Cooperation and Publications, Institute of Public Health of Montenegro, Podgorica, Montenegro; 3 Institute of Chemical, Environmental and Bioscience Engineering, Research Area of Biochemical Technology, TU Wien, Vienna, Austria

**Keywords:** dietary guidelines, dietary intake, EFSA EU Menu, food transition, macronutrients, Montenegro, public health, Western Balkans

## Abstract

**Introduction:**

Understanding population-level dietary intakes is essential for preventing diet-related non-communicable diseases (NCDs) and informing evidence-based nutrition policies. Until recently, Montenegro lacked nationally representative data on food and nutrient intake aligned with European Food Safety Authority (EFSA) standards. This study provides the first comprehensive analysis of energy and macronutrient intake among Montenegrin adults within the EFSA EU Menu framework.

**Methods:**

A cross-sectional dietary survey was conducted between 2017 and 2022 among 1,011 adults aged 18–74 years, using two non-consecutive 24-hour recalls administered by trained interviewers. Foods were classified using the EFSA FoodEx2 system, and nutrient intakes were calculated using national and EFSA food composition databases. Energy and macronutrient intakes were compared with EFSA Dietary Reference Values (DRVs), and differences across sex and age groups were assessed using appropriate parametric and non-parametric tests, accounting for non-normal intake distributions (*p* < 0.05).

**Results:**

Mean daily energy intake was 2,050 ± 670 kcal, with men consuming significantly more energy than women across all age groups. Macronutrient distribution averaged 41 E% carbohydrates, 40 E% fats, and 16 E% proteins, deviating from EFSA recommendations (45–60 E% CHO; 20–35 E% FAT; 0.83 g/kg bw PROT). Grains contributed the largest share of total energy, followed by meat, dairy, and fats, while fruits and vegetables contributed a relatively small share due to their low energy density. Younger adults derived more energy from sweetened foods and beverages, and men showed greater reliance on meat and alcohol.

**Discussion:**

The Montenegrin diet is characterized by high fat and protein intake and low carbohydrate contribution, reflecting Western dietary trends observed in the region. These findings highlight the need for national dietary guidelines promoting balanced macronutrient distribution and increased consumption of plant-based foods to mitigate NCD risks and align with European nutrition and sustainability goals.

## 1 Introduction

Dietary intake plays a fundamental role in maintaining health and preventing non-communicable diseases (NCDs), which account for the majority of morbidity and mortality globally and in Europe (1). Balanced energy and macronutrient intakes are central to healthy diets, yet large cross-national differences persist in both dietary quality and adherence to nutritional guidelines (2, 3). Understanding population-level dietary intakes is therefore critical for shaping public health policies, setting dietary reference values, and designing effective interventions aimed at reducing diet-related disease burden.

In recent decades, European diets have undergone substantial transformation characterized by increased intake of fats, sugars, and processed foods, including a growing contribution of ultra-processed foods, alongside a decline in complex carbohydrates and plant-based foods (4–7). This so-called “food transition” has been particularly evident in post-socialist and upper–middle-income countries of Southeast Europe, where traditional Mediterranean or Balkan dietary habits are being replaced by Westernized consumption models dominated by animal products and energy-dense foods (8–14). In this context, the term food transition is used to describe shifts from traditional to emerging dietary practices, while nutrition transition refers more specifically to the shift toward excess energy intake and diet-related non-communicable diseases. As a result, overweight and obesity have become widespread, and diet-related chronic diseases such as hypertension, diabetes, and dyslipidemia are on the rise across the Western Balkans (9, 11, 15).

To accurately monitor these trends and ensure data comparability across countries, the European Food Safety Authority (EFSA) established the EU Menu project, an initiative to harmonize national food consumption surveys using standardized methodology, including multiple 24-h dietary recalls and FoodEx2 food classification (16). The EU Menu surveys provide high-quality, nationally representative data that serve as the basis for risk assessment, dietary exposure analysis, and public health policy. Within this framework, several Western Balkan countries have completed or are implementing national dietary surveys following EFSA protocols, including Serbia, Croatia, North Macedonia, and Montenegro (17–20).

Despite geographic and cultural similarities, dietary data from the Western Balkans remain limited and fragmented. Existing evidence points to common regional characteristics such as high consumption of meat, dairy, and refined grains, combined with low intake of fruits, vegetables, and dietary fiber (11, 21–24). The overall macronutrient structure typically reveals excessive fat contribution—often exceeding 35% of total energy, and suboptimal carbohydrate intake, indicating deviation from EFSA Dietary Reference Values (3, 11–13). However, national-level analyses remain scarce, and Montenegro has until recently lacked population-based dietary intake data that are methodologically aligned with EU standards.

Montenegro’s population exhibits several risk factors relevant to dietary assessment. According to national health reports, over one third of adults are overweight and nearly one fifth obese, with increasing trends in metabolic syndrome and diet-related conditions (25). Moreover, socioeconomic and geographic disparities, particularly between urban and rural regions and among age groups—may further shape dietary behaviors and nutritional outcomes. Yet, before the implementation of the EFSA EU Menu project (2017–2022), no comprehensive, nationally representative data existed on actual food and nutrient consumption among Montenegrin adults (17).

This study therefore aimed to fill a crucial data gap by providing the first national-level analysis of energy and macronutrient intake in Montenegro based on EFSA’s harmonized methodology. Specifically, it seeks to:

1. Quantify daily energy and macronutrient intake among Montenegrin adults aged 18–74 years.
2. Evaluate the alignment of observed intakes with EFSA Dietary Reference Values (DRV) for macronutrients.
3. Analyze gender- and age-related differences in nutrient intake and food group contributions to total energy.
4. Compare Montenegrin results with data from other Western Balkan and European countries participating in the EFSA EU Menu surveys.

By providing comparable, evidence-based dietary intake data, this study contributes to the regional understanding of food transition patterns and supports the formulation of national dietary guidelines in Montenegro. The results are expected to inform public health strategies targeting the prevention of diet-related NCDs and to strengthen Montenegro’s participation in the broader European food consumption monitoring network.

## 2 Materials and methods

### 2.1 Study design and population

The Montenegrin National Dietary Survey was conducted within the framework of the EFSA EU Menu project between 2017 and 2022, following the harmonized guidance for national food consumption surveys (16). The study applied a cross-sectional design to collect nationally representative dietary data among adults aged 18–74 years residing in all three Montenegrin regions (Northern, Central, and Coastal).

Sampling was based on a stratified, multistage design developed according to Eurostat and EFSA recommendations, ensuring proportional representation by sex, age group, and region. A total of 1,011 individuals were recruited, with 48.2% men and 51.8% women. The sample size was determined to achieve sufficient statistical power for subgroup analyses and to allow direct comparability with other EU Menu surveys.

Eligible participants were permanent residents of Montenegro who were cognitively and physically capable of completing two 24-h dietary recalls. Exclusion criteria included pregnancy, lactation, serious illness, or inability to provide reliable dietary information. Written informed consent was obtained from all participants. The study protocol was approved by the Ethics Committee of the University of Donja Gorica (Approval No. UDG-NS-2017/3) and followed the principles of the Declaration of Helsinki.

### 2.2 Data collection

Data collection was carried out by trained nutritionists and field interviewers. Each participant completed two non-consecutive 24-h dietary recalls covering both weekdays and weekends, spaced at least 2 weeks apart to capture day-to-day variability. Interviews were conducted face-to-face using the DIET ASSESS & PLAN (DAP) software, harmonized with the EFSA EU Menu specifications (26). The system integrates standardized probing questions and portion size estimation tools consistent with EFSA guidance.

To enhance accuracy, food portion sizes were estimated using photographic atlases, household measures, and standard serving utensils validated for the Montenegrin context. Participants were instructed to report all foods, beverages, and supplements consumed during the preceding 24 h, including preparation methods and brand names where applicable. Alcoholic beverages were recorded as part of the 24-h dietary recalls and coded within the FoodEx2 beverage categories, in accordance with EFSA EU Menu guidance. As in other dietary surveys, alcohol intake may be subject to underreporting due to social desirability bias.

Socio-demographic and lifestyle data—including age, sex, education, employment status, smoking, physical activity, and self-reported health—were collected via structured questionnaires harmonized with the EFSA guidance (16). Anthropometric measurements (body height and weight) were obtained using standardized protocols and calibrated instruments, and body mass index (BMI) was calculated as kg/m^2^.

### 2.3 Food classification and nutrient composition

All reported foods were coded using the EFSA FoodEx2 hierarchical classification system (version 2.0), which enables standardized categorization of food items across EU Member States (27, 28). Each reported food item was linked to nutrient composition data from the Montenegrin Food Composition Database, supplemented where necessary by the EFSA European Food Composition Database and cross-checked against the West Balkan Food Composition Database (12). For analytical purposes, individual FoodEx2 codes were aggregated into 13 major food groups (beverages [non-milk], eggs and egg products, fats and oils, fruits and fruit products, grains and grain products, meat and meat products, milk and milk products or substitutes, miscellaneous food products, nuts/seeds and kernel products, products for special nutritional use or dietary supplements, seafood and related products, sugars and sugar products, and vegetables and vegetable products). For analyses of dietary energy contribution, these food groups were used consistently to calculate the percentage contribution to total energy intake (%TE) and to enable comparisons across tables and figures. Nutrient composition data were primarily derived from the Montenegrin Food Composition Database, developed within the Balkan Food Platform and harmonized with EuroFIR standards. The database contained more than 2,000 food items and over 300 traditional and composite recipes representative of the Western Balkan diet. Both raw and cooked foods are included, with nutrient values for prepared dishes calculated using standardized recipes, yield factors, and nutrient retention factors in accordance with EuroFIR and EFSA recommendations (29).

Energy and nutrient values were calculated using standard conversion factors: 4 kcal/g for carbohydrates and protein, and 9 kcal/g for fat (30). For mixed dishes and composite meals, nutrient values were estimated based on ingredient proportions and cooking yields recorded during dietary interviews.

### 2.4 Dietary data processing and quality control

Dietary data were processed and validated using the DIET ASSESS and PLAN (DAP) software, developed by the Centre of Research Excellence in Nutrition and Metabolism, Institute for Medical Research, University of Belgrade (26). Body weight was used to derive energy intake per kilogram of body weight (kcal/kg bw/day) for standardized energy intake analyses.

The DAP platform is harmonized with EFSA EU Menu requirements and incorporates standardized modules for FoodEx2 food coding, nutrient calculation, quality control, and plausibility checks equivalent to those implemented in EFSA’s Dietary Assessment Software Platform (DASP) (26).

Reported energy and nutrient intakes were examined for completeness, outliers, and potential misreporting using Goldberg cut-offs (BMR × 1.1–2.5). Cases with implausible energy intakes relative to body weight or physical activity level were reviewed individually by trained assessors.

Quality control was maintained through several procedures, including:

- Standardized interviewer training and certification;
- Double data entry for 10% of records;
- Cross-validation of food composition data sources;
- Internal audits following EFSA’s Quality Assurance Plan (16).

Discrepancies identified during double data entry were minimal and were resolved through verification against original questionnaires by trained assessors prior to final data analysis.

### 2.5 Statistical analysis

Statistical analyses were performed using IBM SPSS Statistics (v28) and R (v4.3.1). Descriptive statistics were calculated for continuous variables (means, standard deviations, medians, minimum and maximum values) and categorical variables (percentages and frequency distributions). Normality was assessed using the Shapiro–Wilk test. Given the non-normal distribution of energy and macronutrient intake variables, results are presented using both parametric (mean ± SD) and non-parametric (percentiles) summary statistics, including the 5th, 25th, 50th, 75th, and 95th percentiles.

Comparisons between men and women, and across age groups (18–24, 25–44, 45–64, 65–74 years), were conducted using independent-samples *t*-tests or Mann–Whitney *U* tests for two groups, and one-way ANOVA or Kruskal–Wallis tests for multiple groups, depending on data distribution. Post-hoc analyses with Bonferroni correction were applied where appropriate. Sex-specific comparisons within age groups were conducted using the Mann–Whitney U test due to skewed intake distributions.

Macronutrient energy contributions were calculated as percentages of total energy intake (%E) and compared with EFSA Dietary Reference Values (3). Energy contributions by food group were derived as median percentages of total energy (%TE) from each FoodEx2 major food group. Differences in energy contributions by sex and age group were assessed using nonparametric tests.

In addition, macronutrient density was expressed as grams per 1,000 kilocalories (g/1000 kcal), and energy intake was standardized per kilogram of body weight (kcal/kg bw/day), to enable comparisons independent of total energy intake and body size.

To estimate the probability of inadequate (or excessive) intake relative to EFSA reference values, intake distributions were normalized using Box–Cox transformation, and probabilities of intake below or above the corresponding cut-off points were derived from the fitted distributions.

Age- and sex-adjusted estimates of percentage contribution to total energy intake (%TE) were obtained using regression models including age and sex as covariates, with corresponding 95% confidence intervals. To explore potential inverse causality and selective underreporting of energy intake, an exploratory linear regression analysis was performed with reported daily energy intake (kcal/day) as the dependent variable and body weight (kg) as the independent variable. This analysis was conducted for descriptive purposes only, acknowledging the limitations of cross-sectional 24-h dietary recall data for causal inference.

All statistical tests were two-tailed, with a significance level set at *p* < 0.05. Data visualization was performed using appropriate statistical software in R and Python.

## 3 Results

The results section presents the demographic characteristics, macronutrient intakes, and energy contributions by food groups, with comparisons to EFSA reference values.

### 3.1 Population characteristics of the Montenegrin sample

**Figure 1:**
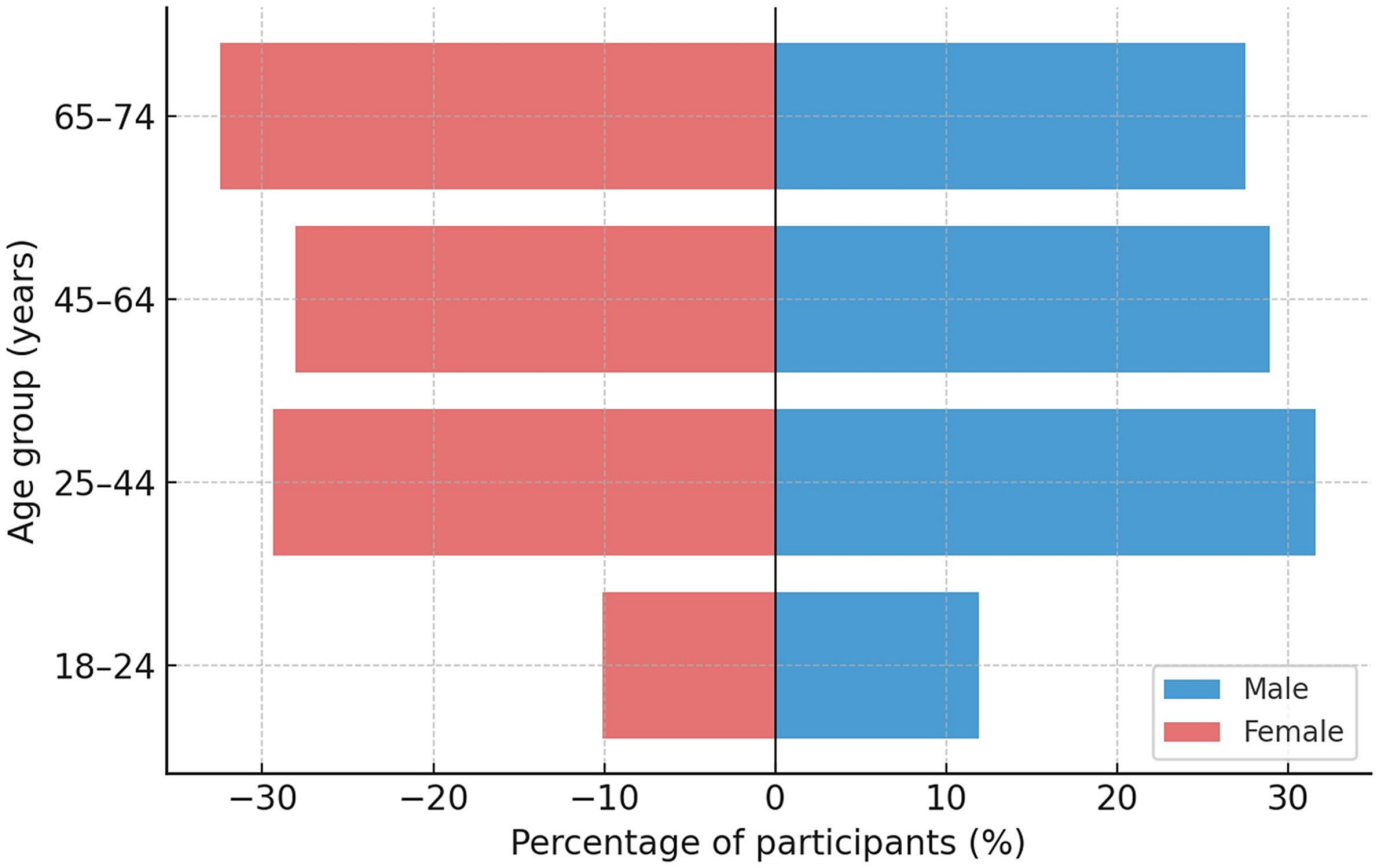
Age and sex distribution of Montenegrin adults participating in the EFSA EU Menu national dietary survey (2017–2022) (*N* = 1,011).

A total of 1,011 adults aged 18–74 years participated in the Montenegrin National Dietary Survey conducted within the EFSA EU Menu framework (2017–2022) (17). The sample included 48.2% men and 51.8% women and was geographically and demographically representative of the national population (Table 1). The age and sex distribution is illustrated in Figure 1, showing a balanced structure across age groups, with the largest share among individuals aged 45–64 years (34.1%) and the smallest among those aged 65–74 years (13.8%). Regional distribution mirrored the population structure: 42.5% of participants were from the central, 31.7% from the northern, and 25.8% from the coastal region. A higher proportion of participants lived in urban areas (61.2%) compared to rural ones (38.8%).

**Table 1:** Demographic, anthropometric, and lifestyle characteristics of Montenegrin adults (*N* = 1,011).

Variable	Category	Total (%)	Men (%)	Women (%)	*p*-value
Age group (years)	18–24	22.6	21.3	23.8	0.353
	25–44	29.5	30.4	28.7	
	45–64	34.1	34.9	33.3	
	65–74	13.8	13.4	14.2	
Region	Central	42.5	42.9	42.1	0.912
	Northern	31.7	31.2	32.1	
	Coastal	25.8	25.9	25.8	
Settlement	Urban	61.2	62.4	60.1	0.184
	Rural	38.8	37.6	39.9	
Education	Primary	16.9	14.1	19.5	<0.001
	Secondary	47.3	50.1	44.6	
	Tertiary	35.8	35.8	35.9	
Labor status	Employed	56.4	60.8	52.3	<0.001
	Unemployed	18.7	15.3	21.9	
	Retired	15.9	15.2	16.6	
	Student	9.0	8.7	9.2	
BMI (kg/m^2^)	Mean ± SD	25.5 ± 4.1	26.9 ± 3.7	24.2 ± 3.9	<0.001
BMI categories	Underweight	4.7	2.8	6.4	<0.001
	Normal weight	47.5	43.2	51.4	
	Overweight	33.6	37.1	30.4	
	Obese	14.2	16.9	11.8	
Physical activity	Low	32.1	28.4	35.5	<0.001
	Moderate	46.3	45.8	46.7	
	High	21.6	25.8	17.8	
Smoking status	Current	33.9	39.6	28.6	<0.001
	Former	19.4	21.0	18.0	
	Never	46.7	39.4	53.4	
Special diet	Normal diet	75.8	79.1	72.5	<0.001
	Slimming diet	6.2	4.8	7.4	
	Diet related to health conditions (e.g., diabetes, allergy, celiac)	10.8	10.2	11.3	
	Vegetarian diet	3.5	2.7	4.2	
	Unclassified	3.7	3.2	4.6	
Health condition	Normal condition	79.6	81.2	78.1	<0.001
	Chronic/long-term disease	18.9	17.5	20.2	
	Unclassified	1.5	1.3	1.7	

Values are presented as percentages (%) for categorical variables and as mean ± standard deviation (SD) for continuous variables. Differences between men and women were assessed using the Chi-square test for categorical variables (age group, region, settlement, education, labor status, BMI categories, physical activity level, smoking status, special diet, and health condition) and the Mann–Whitney *U* test for continuous variables (BMI, kg/m^2^), due to non-normal distribution of the data.

A *p*-value < 0.05 was considered statistically significant.

Educational attainment and employment patterns were consistent with national statistics: 47.3% of respondents had secondary education, 35.8% tertiary, and 16.9% primary. More than half were employed (56.4%), while 18.7% were unemployed, 15.9% retired, and 9.0% students. The majority were married or cohabiting (63.1%), 22.8% single, and 14.1% widowed or divorced.

**Figure 2:**
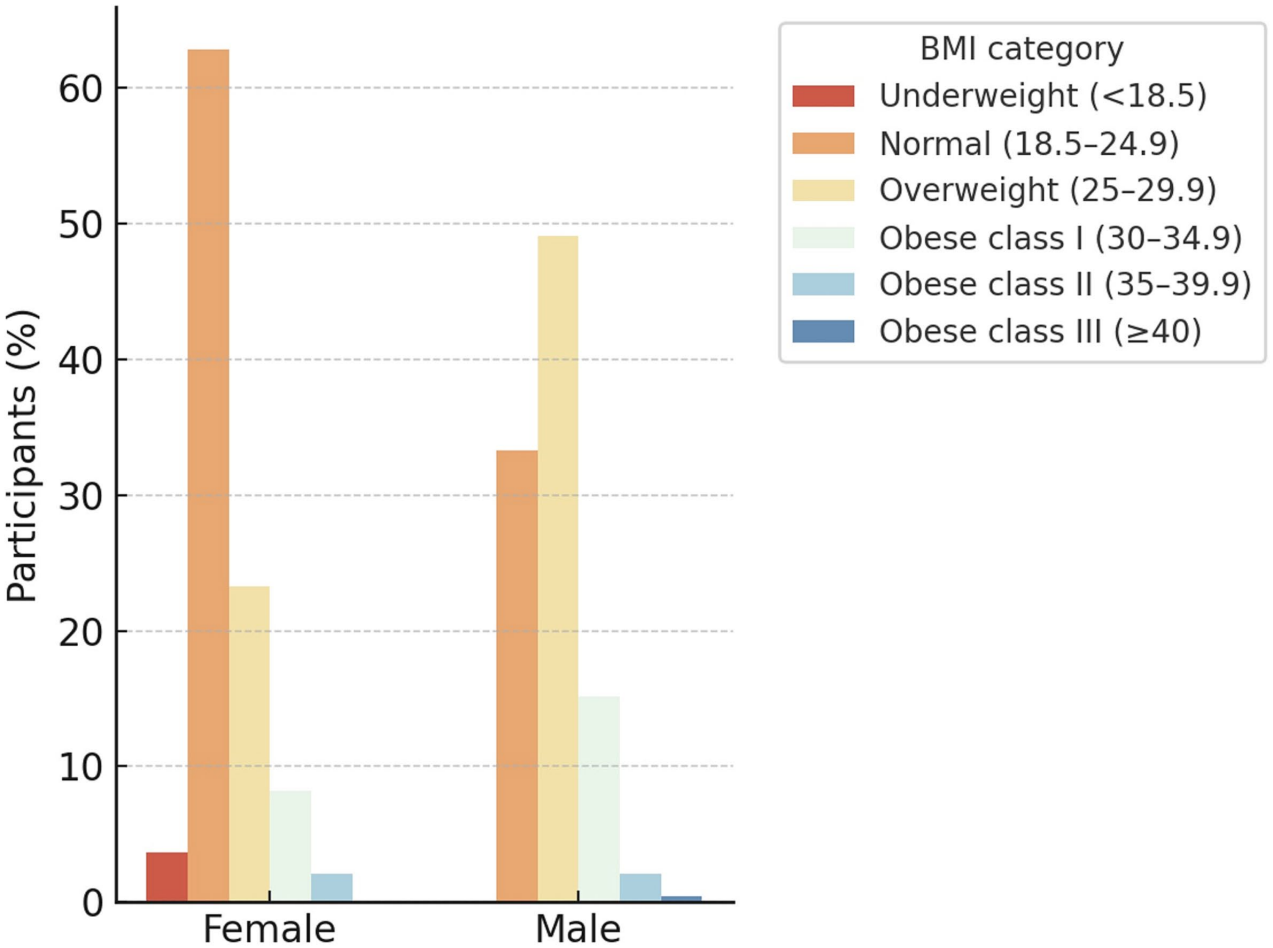
Distribution of BMI categories by gender according to WHO classification (*N* = 1,011).

Average body height was 174.3 ± 8.1 cm in men and 163.2 ± 7.2 cm in women, while body weight averaged 80.8 ± 11.4 kg and 64.3 ± 9.8 kg, respectively. The mean BMI for the total sample was 25.5 ± 4.1 kg/m^2^, with significantly higher values among men (26.9 ± 3.7 kg/m^2^) than women (24.2 ± 3.9 kg/m^2^, *p* < 0.001). According to the WHO classification, 47.5% of adults had normal weight, 33.6% were overweight, 14.2% obese, and 4.7% underweight (Figure 2). The prevalence of overweight and obesity was notably higher among men, whereas women more often fell within the normal-weight range.

Self-reported lifestyle indicators showed that 46.3% of participants engaged in moderate physical activity, 32.1% in low, and 21.6% in high activity levels. Men were more often highly active (25.8%) compared to women (17.8%). Smoking was reported by 33.9% of adults (39.6% of men and 28.6% of women), and 21.4% declared following a special diet. Approximately 38.6% reported at least one chronic health condition, most frequently hypertension (14.8%) or diabetes (7.2%).

### 3.2 Macronutrient density and energy intake standardized for body size

**Figure 3:**
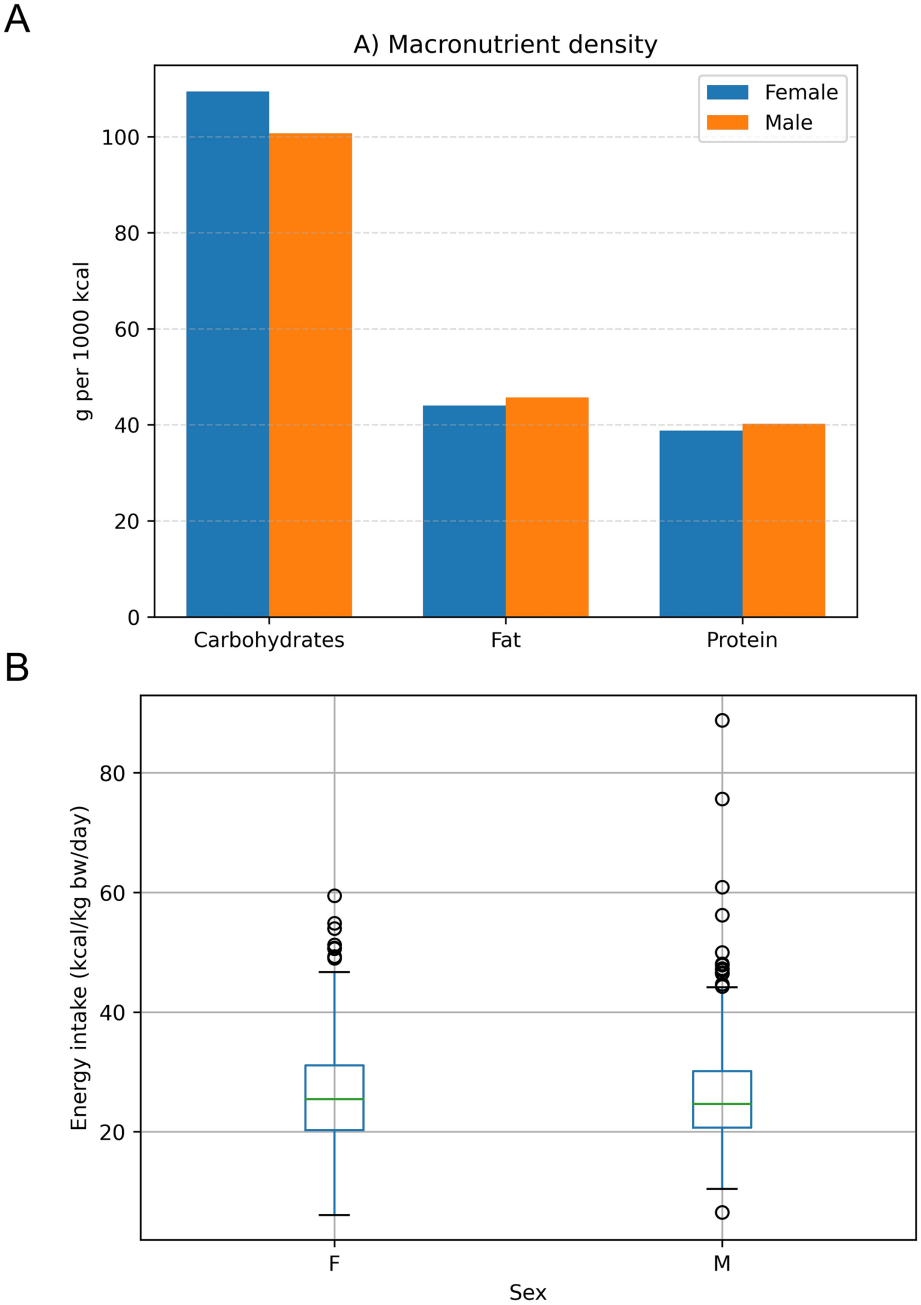
Macronutrient density and energy intake standardized for body size (*N* = 1,011). **(A)** Macronutrient density expressed as grams per 1,000 kilocalories (g/1000 kcal), by sex. **(B)** Daily energy intake expressed per kilogram of body weight (kcal/kg bw/day), by sex (boxplots show median and interquartile range).

To allow comparison of dietary composition independent of total energy intake, macronutrient density was expressed as grams per 1,000 kilocalories (g/1000 kcal) (Figure 3A). Carbohydrates represented the highest nutrient density, followed by fat and protein, with similar patterns observed between men and women. When energy intake was standardized per kilogram of body weight (kcal/kg bw/day), no substantial differences were observed between men and women (Figure 3B), indicating comparable energy intake relative to body size despite higher absolute energy intake among men.

### 3.3 Energy and macronutrient intake

The mean daily energy intake was 2,050 ± 670 kcal, while the mean intakes of macronutrients were 212.8 ± 74.5 g carbohydrates (CHO), 93.1 ± 37.6 g fat (FAT), and 81.0 ± 30.0 g protein (PROT) (Table 1). Men exhibited substantially higher energy and macronutrient intakes compared with women across all age groups (Table 2). The highest mean energy intake was recorded among men aged 18–24 years (2,543 ± 861 kcal/day), while the lowest intake was observed among women aged 45–64 years (1,758 ± 491 kcal/day). Across all age groups, the macronutrient distribution indicated a diet rich in fats and proteins but relatively low in carbohydrates (Table 3). The differences between men and women were statistically significant for all macronutrients (*p* < 0.001). Due to the skewed distribution of intake variables, detailed distributional characteristics including percentiles (P5–P95) are presented in Table 2.

**Table 2:** Descriptive statistics of energy and macronutrient intakes among Montenegrin adults (*N* = 1,011).

Variable	Mean ± SD	P5	P25	P50 (median)	P75	P95	Normality test *p*-value
Energy (kcal/day)	2050.4 ± 669.7	1134.3	1585.2	1983.4	2416.0	3248.5	<0.001
Carbohydrates (g/day)	212.8 ± 74.5	110.6	165.1	203.6	250.4	337.0	<0.001
Fat (g/day)	93.1 ± 37.6	40.3	65.7	87.8	115.0	161.1	<0.001
Protein (g/day)	80.4 ± 28.8	41.6	59.7	77.0	96.2	132.7	<0.001

Percentiles (P5–P95) are presented to describe intake distributions.

Normality was assessed using the Shapiro–Wilk test.

All variables showed significant deviation from normality (*p* < 0.001).

**Table 3:** Energy and macronutrient intakes by sex and age group (*N* = 1,011).

Age group (years)	Variable	Men (mean ± SD)	Women (mean ± SD)	*p*-value
18–24	Energy (kcal/day)	2542.9 ± 861.3	1852.8 ± 461.9	<0.001
	Carbohydrates (g/day)	263.1 ± 110.4	211.8 ± 61.3	0.005
	Fat (g/day)	118.0 ± 40.0	80.3 ± 29.4	<0.001
	Protein (g/day)	99.9 ± 31.6	67.1 ± 23.6	<0.001
	Protein (g/kg bw/day)	1.2 ± 0.4	1.1 ± 0.4	0.027
25–44	Energy (kcal/day)	2411.3 ± 645.1	1813.5 ± 520.2	<0.001
	Carbohydrates (g/day)	241.2 ± 75.7	192.7 ± 61.7	<0.001
	Fat (g/day)	112.9 ± 36.5	82.6 ± 29.5	<0.001
	Protein (g/day)	95.8 ± 29.5	68.2 ± 20.8	<0.001
	Protein (g/kg bw/day)	1.1 ± 0.3	1.0 ± 0.3	0.730
45–64	Energy (kcal/day)	2255.9 ± 697.2	1757.8 ± 491.3	<0.001
	Carbohydrates (g/day)	222.9 ± 74.5	193.5 ± 59.4	<0.001
	Fat (g/day)	102.7 ± 39.2	76.5 ± 28.6	<0.001
	Protein (g/day)	90.1 ± 29.7	68.6 ± 22.5	<0.001
	Protein (g/kg bw/day)	1.0 ± 0.3	0.9 ± 0.3	0.394
65–74	Energy (kcal/day)	2232.6 ± 703.3	1770.4 ± 512.2	<0.001
	Carbohydrates (g/day)	223.2 ± 79.3	188.3 ± 60.2	<0.001
	Fat (g/day)	102.1 ± 40.4	79.7 ± 32.9	<0.001
	Protein (g/day)	89.0 ± 29.1	70.3 ± 22.9	<0.001
	Protein (g/kg bw/day)	1.0 ± 0.3	1.0 ± 0.3	0.902

Values are presented as mean ± standard deviation. Differences between men and women within each age group were assessed using the Mann–Whitney *U* test due to non-normal intake distributions.

### 3.4 Comparison with EFSA dietary reference values

**Figure 4:**
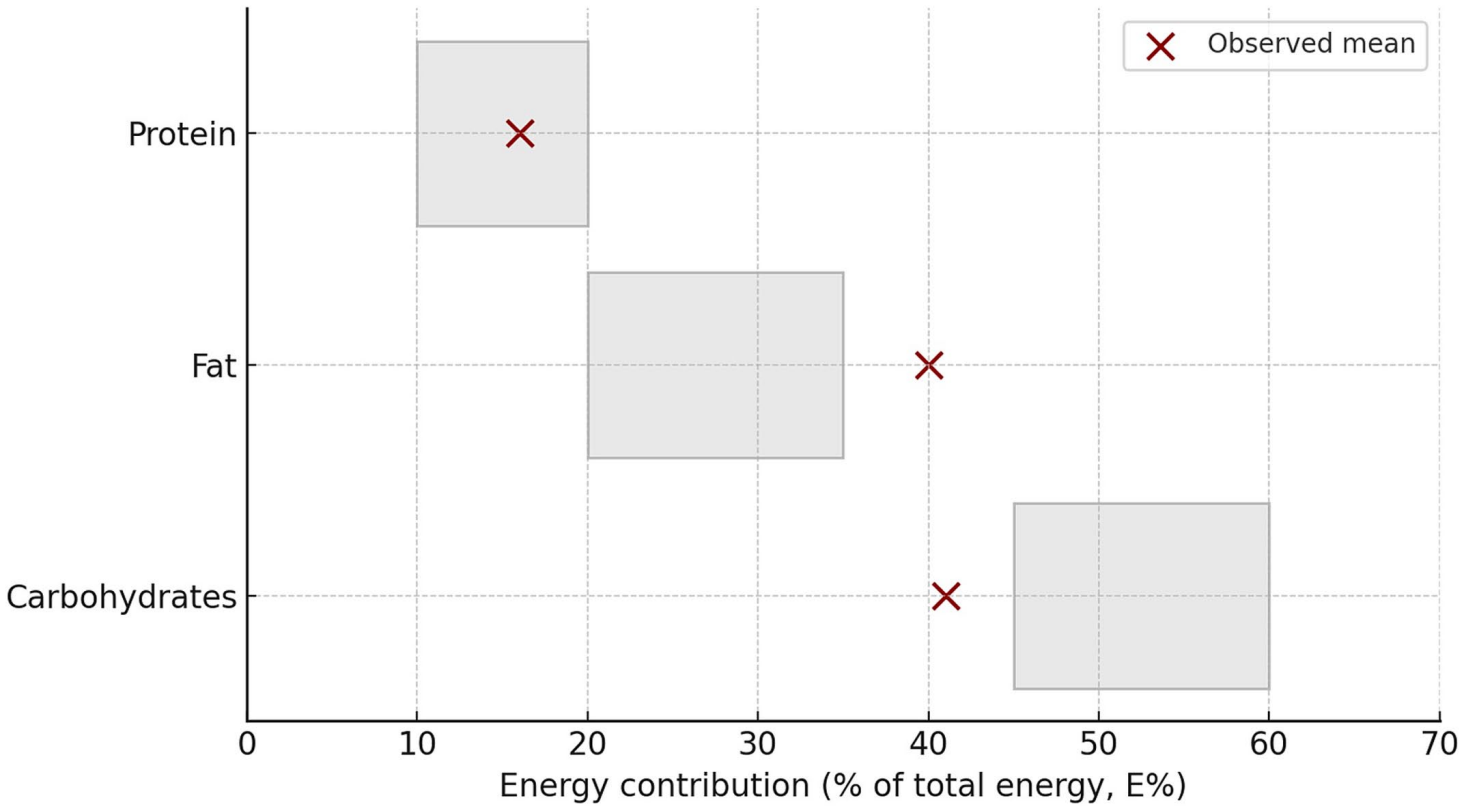
Macronutrient energy contribution (E%) among Montenegrin adults compared with EFSA recommended ranges (RI) (*N* = 1,011).

The observed energy and nutrient intakes were compared with the European Food Safety Authority (EFSA) Dietary Reference Values (DRVs) for adults (Table 4). In addition to descriptive comparisons, the probability of inadequate or excessive intake relative to EFSA reference values was estimated using normalized intake distributions, allowing assessment of intake adequacy given the non-normal distribution of dietary variables (Figure 4).

**Table 4:** Probability of inadequate (or excessive) intake relative to EFSA cutoff values, estimated from normalized intake distributions.

Indicato	EFSA cutoff	Reference type	Direction	Probability (%)
Carbohydrates (E%)	45 E%	RI (lower bound)	Below cutoff	**63.3**
Fat (E%)	35 E%	RI (upper bound)	Above cutoff	**75.7**
Protein (g/kg bw/day)	0.83 g/kg bw/day	PRI	Below cutoff	**31.5**

Probabilities were derived from Box–Cox–normalized intake distributions to account for skewed intake data. Macronutrient energy contribution was calculated as: CHO_E% = (CHO_g × 4/ENERC_kcal) × 100; FAT_E% = (FAT_g × 9/ENERC_kcal) × 100. Protein adequacy was evaluated as PROT_g_per_kg = PROT_g/body weight (kg). Bold values indicate the estimated proportion (%) of the population with inadequate or excessive intake relative to EFSA cutoff values.

The mean energy intake among men (2,233–2,543 kcal) was within the EFSA reference range for moderately active males (2,290–2,670 kcal), whereas women’s mean intake (1,758–1,853 kcal) corresponded to the lower end of the EFSA range (1,860–2,150 kcal).

However, the macronutrient energy distribution deviated from EFSA recommendations: carbohydrate intake contributed ≈ 41 E%, below the recommended 45–60 E%; fat contributed ≈ 40 E%, exceeding the upper EFSA limit (35 E%); and protein intake contributed ≈ 16 E%, slightly above the population reference intake (0.83 g/kg bw/day).

### 3.5 Distribution of total energy intake

The overall distribution of energy intake showed considerable variability, with a long right tail representing individuals with high daily energy consumption.

**Figure 5:**
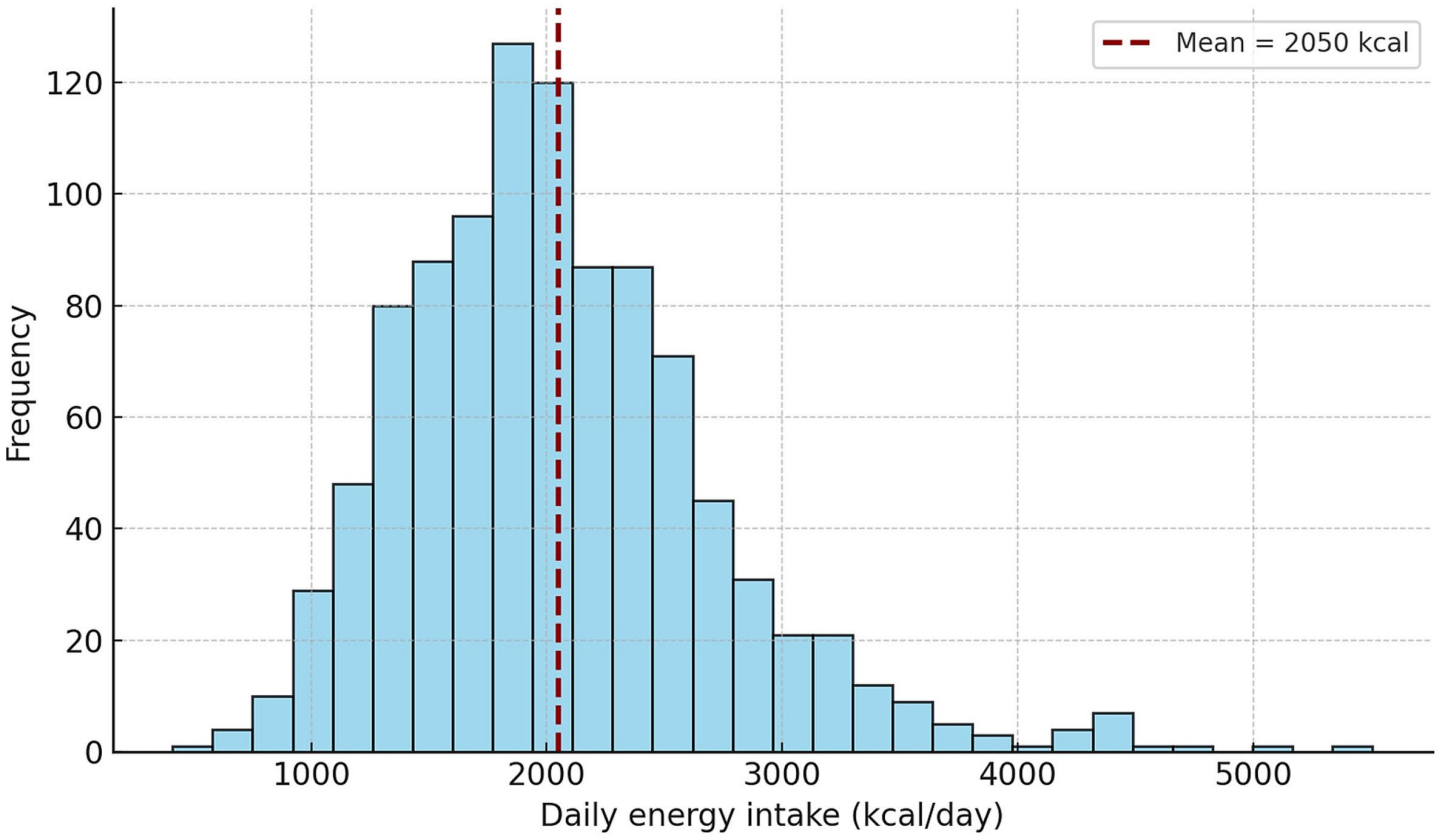
Distribution of daily energy intake among Montenegrin adults (*N* = 1,011).

The mean intake (2,050 kcal/day) was close to the median (1,983 kcal/day), suggesting a slightly right-skewed distribution typical for population dietary surveys (Figure 5). Exploratory regression analysis showed a statistically significant but weak association between reported energy intake and body weight (*β* ≈ 15.6 kcal/kg, *R*^2^ = 0.15), suggesting potential underestimation of energy intake among individuals with higher body weight.

### 3.6 Energy contribution by food groups

The contribution of individual food groups to total energy intake was calculated to assess the structure of the Montenegrin diet and identify key sources of energy among adults aged 18–74 years. Table 5 presents the median percentage of total energy intake (%TE) provided by each major food group. In addition to crude estimates, age- and sex-adjusted mean %TE values with corresponding 95% confidence intervals are reported to account for demographic differences.

**Table 5:** Contribution of food groups to total energy intake (%TE) with 95% confidence intervals.

Food group	Raw mean %TE (95% CI)	Age- and sex-adjusted mean %TE (95% CI)
Grains and grain products	**32.6** (31.9–33.2)	**32.6** (31.9–33.2)
Meat and meat products	**15.8** (15.2–16.3)	**15.7** (15.2–16.3)
Milk and milk products (including substitutes)	**14.6** (14.2–15.1)	**14.6** (14.2–15.1)
Fats and oils	**14.4** (14.0–14.8)	**14.4** (14.0–14.8)
Vegetables and vegetable products	**7.8** (7.5–8.1)	**7.8** (7.5–8.1)
Fruits and fruit products	**7.8** (7.4–8.2)	**7.7** (7.4–8.1)
Sugar and sweets	**6.3** (5.9–6.7)	**6.2** (5.9–6.6)
Beverages (non-milk)	**4.7** (4.3–5.0)	**4.5** (4.2–4.9)

Bold values indicate mean percentage contributions to total energy intake (%TE). Values in parentheses represent 95% confidence intervals.

**Figure 6:**
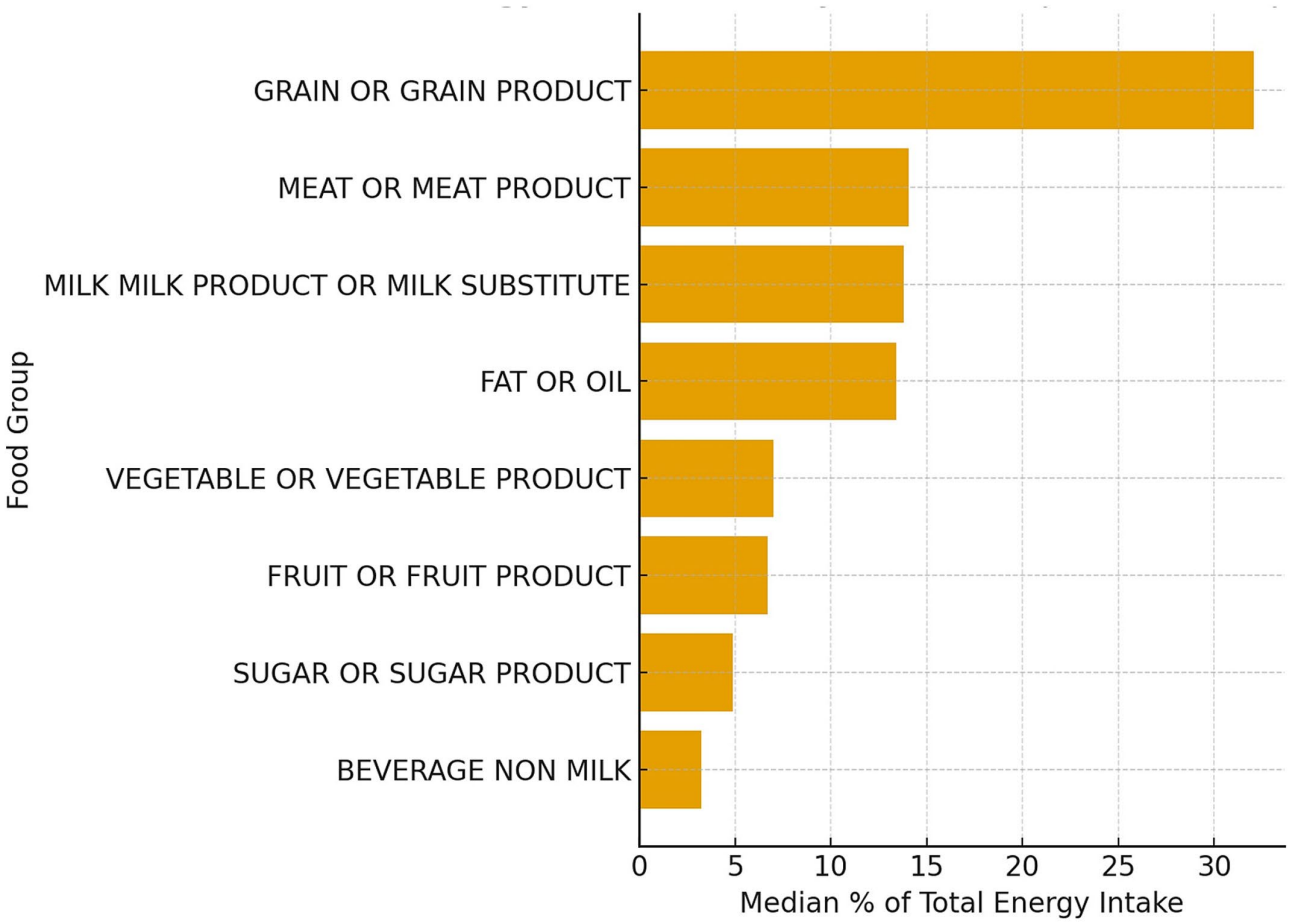
Median percentage of total energy intake from each food group in the total sample (*N* = 1,011).

Overall, grains and grain products represented the largest share of total energy intake (~32% TE), followed by meat and meat products (14%), milk and dairy (14%), and fats and oils (13%). Fruits and vegetables together contributed approximately 14% of total energy, whereas sugars and beverages accounted for less than 10% (Figure 6).

### 3.7 Gender differences in energy contribution

Table 6 summarizes the energy contribution of different food groups by gender, together with statistical significance (Mann–Whitney *U* test). Results are presented as median %TE values due to skewed distributions.

**Table 6:** Sex- and age-related differences in the median contribution of food groups to total energy intake.

Food group	♀ Median %TE	♂ Median %TE	*p* (gender)	*p* (age group)
Grains/grain products	**33.3**	**31.0**	**<0.001**	0.29
Meat/meat products	**12.6**	**15.4**	**<0.001**	0.61
Milk/milk products	13.6	13.8	0.67	0.07
Fat/oils	13.4	13.4	0.72	0.10
Vegetables	6.9	7.1	0.21	0.35
Fruits	**7.9**	**5.4**	**<0.001**	0.24
Sugar/sweets	5.5	4.5	0.07	**<0.001**
Beverages (non-milk)	**2.5**	**3.8**	**<0.001**	**0.03**

Values are presented as median percentage of total energy intake (%TE). Differences between men and women were assessed using the Mann–Whitney *U* test, while differences across age groups were assessed using the Kruskal–Wallis test. Median values were used due to the skewed distribution of food group energy contributions. Bold values indicate statistically significant differences (*p* < 0.05) between groups.

**Figure 7:**
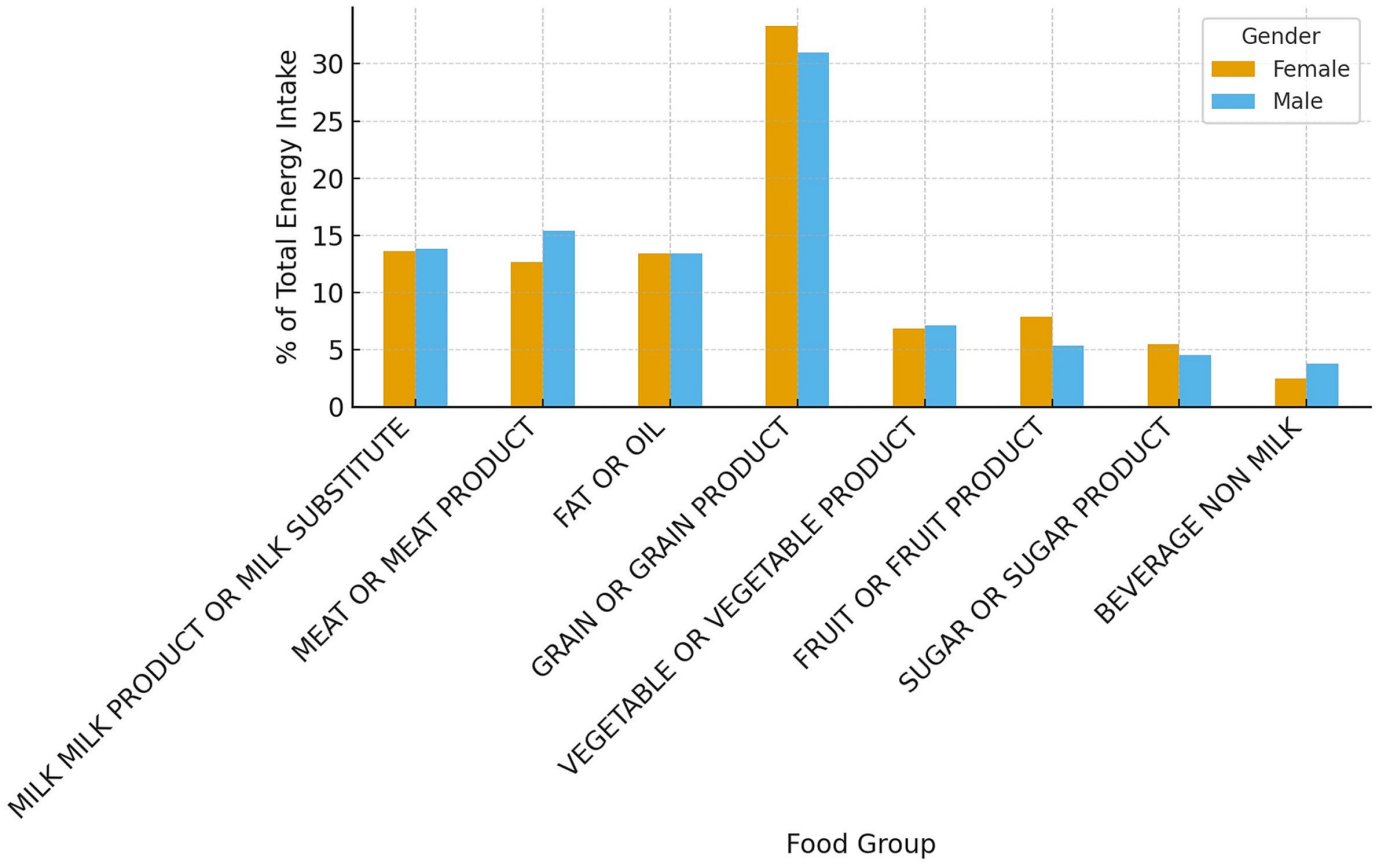
Comparison of energy contributions by food group and gender (*N* = 1,011).

Women derived a significantly greater proportion of their daily energy from grains (*p* < 0.001), fruits (*p* < 0.001), and sugars (trend: *p* = 0.07), while men obtained a higher share from meat (*p* < 0.001) and beverages (*p* < 0.001). The contribution of milk and fats was similar between genders (Figure 7).

### 3.8 Age-related differences

**Figure 8:**
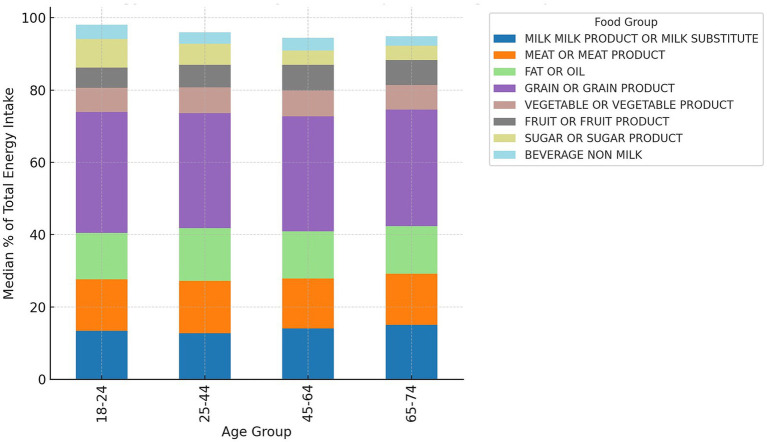
Distribution of %TE from food groups across age groups (18–24, 25–44, 45–64) (*N* = 1,011).

Kruskal–Wallis testing revealed statistically significant differences in energy contributions of sugars (*p* < 0.001) and beverages (*p* = 0.03) across age groups. Younger adults (18–24 years) derived a higher percentage of total energy from sweetened foods and beverages compared to older participants, while older adults (45–64 years) obtained relatively more energy from milk and fats (Figure 8). These age-related differences remained consistent with the overall dietary pattern observed in the total sample.

## 4 Discussion

### 4.1 Overview of main findings

This nationally representative EFSA EU Menu survey provides the first comprehensive assessment of energy and macronutrient intake among Montenegrin adults. Mean daily energy intake was 2,050 kcal, with men consuming substantially more energy and macronutrients than women. The observed macronutrient distribution, characterized by a high contribution from fats (≈40 E%), moderate protein intake (≈16 E%), and a low contribution from carbohydrates (≈41 E%), deviated from EFSA Dietary Reference Values. In addition, the dietary energy profile was dominated by grains, meat, dairy, and added fats, consistent with a high-fat pattern and a relatively low carbohydrate contribution. While fruits and vegetables contributed a small share of total energy, as expected given their low energy density, this metric should not be interpreted as an indicator of adequacy; therefore, fruit and vegetable intake is discussed in relation to dietary recommendations rather than energy contribution. Collectively, these findings point to a pronounced food transition toward energy-dense, predominantly animal-based and processed foods, consistent with trends reported across the Western Balkans (3, 9, 31, 32). Future studies using the same nationally representative database may apply dietary pattern–based approaches to further characterize eating behaviors beyond macronutrient distributions. These patterns remained consistent when macronutrient intake was expressed relative to total energy intake and body size.

### 4.2 Comparison with European and regional data

The macronutrient distribution observed among Montenegrin adults (≈41 E% carbohydrates, ≈40 E% fats, ≈16 E% proteins) is highly consistent with other Western Balkan findings. In Serbia, the national EFSA EU Menu survey reported mean contributions of 41.5 E% carbohydrates, 39.2 E% fats, and 16.3 E% proteins in adults aged 18–64 years (9). Similarly, data from North Macedonia show a fat-rich energy structure dominated by animal-based foods and refined grains, confirming a broader regional trend toward energy-dense diets (33).

Compared with Mediterranean EU countries, Montenegrin adults obtain less energy from carbohydrates and more from total fat. Systematic reviews of national dietary surveys compiled in the EFSA Comprehensive European Food Consumption Database indicate that Spain, Italy, and Greece typically exhibit higher carbohydrate shares (≈47–53 E%) and lower total fat shares (≈33–37 E%), consistent with Mediterranean dietary characteristics (34). For example, the Spanish ANIBES study reported a national average of ≈41 E% carbohydrates, 38.5 E% fats, and 16.8 E% proteins, showing a more balanced distribution than that found in Western Balkan populations (35). The Greek HYDRIA survey likewise confirmed relatively higher carbohydrate intake and lower saturated-fat contribution among adults (36). In France, national INCA3 data place adult fat contributions at approximately 35 E%, again below the ≈40 E% observed in Montenegro (37).

Taken together, these comparisons situate Montenegro within a Western Balkan macronutrient pattern characterized by high fat and low carbohydrate intake, diverging from the Mediterranean EU profile.

This east-to-west gradient in dietary structure has been repeatedly highlighted in European nutritional assessments, which note that Balkan and Eastern European populations show faster transitions toward energy-dense, animal-based, and processed-food consumption patterns than their Mediterranean counterparts (34, 36, 37).

### 4.3 Gender- and age-related differences in dietary intake

Gender and age differences were evident: men consumed significantly more total energy and derived a higher relative energy contribution from meat and beverages, while women derived a greater proportion of energy from grains and plant-based foods. Younger adults exhibited higher consumption of sugars and sweetened beverages, whereas older groups derived more energy from dairy and fats. These patterns are in line with findings reported across European populations. Men generally report higher absolute energy and fat intakes, while women derive a greater proportion of energy from carbohydrates and plant-based foods. Comparable findings were demonstrated in the UK Biobank cohort, where men consumed significantly more total energy, fats, and proteins than women, whereas women had higher relative carbohydrate contributions and better adherence to dietary recommendations for fruit and vegetable intake (38).

Similar patterns were confirmed in the Lithuanian National Nutrition Survey (2019–2020), which revealed that men and older adults obtained a larger share of energy from fats and alcohol, while women and younger participants showed relatively higher carbohydrate proportions (39). These differences were also associated with physical activity levels and occupational status, suggesting lifestyle-driven variation in macronutrient distribution.

In Southern Europe, Italian data likewise highlight gender- and age-related disparities: men tend to favor meat, dairy, and alcoholic beverages, whereas women report greater consumption of fruits, vegetables, and dairy substitutes, often linked to higher health awareness (40). Such differences are consistent with Montenegrin findings, where male participants obtained more energy from meat and beverages, and women derived greater proportions from grains, fruits, and sweets.

Across Europe, these observations support the notion that sex and age are strong determinants of dietary behavior, reflecting sociocultural norms, metabolic demands, and exposure to food marketing. Understanding these distinctions is essential for tailoring public health interventions that promote balanced nutrition across demographic groups.

### 4.4 Public health implications

The high fat and protein but low carbohydrate intake observed in Montenegro may contribute to the growing prevalence of diet-related non-communicable diseases (NCDs), including cardiovascular diseases, diabetes, and obesity. Approximately 38.6% of surveyed adults reported at least one chronic condition, most frequently hypertension or diabetes, consistent with national epidemiological data. These trends align with the broader regional challenge of unhealthy diets and rising overweight rates (1, 10, 12, 21, 33, 41).

The observed low contribution of fruits and vegetables to total energy intake (~14%), reflecting their low energy density, suggests suboptimal absolute consumption when evaluated against WHO recommendations and Mediterranean dietary benchmarks (34). These findings remained robust after adjustment for age and sex, indicating that the observed dietary imbalances are not solely driven by demographic structure. Given that nearly half of adults have a BMI above the normal range, dietary interventions are urgently needed. Implementing culturally adapted dietary guidelines emphasizing whole grains, legumes, fruits, vegetables, and unsaturated fats would align with the European “Farm to Fork” and “Sustainable Healthy Diets” frameworks.

Strengthening national Food-Based Dietary Guidelines (FBDGs) to incorporate both health and sustainability principles—as discussed in Balkan nutrition surveillance and data-harmonization studies (12) would improve population-level nutrition literacy and support evidence-based public health policy.

### 4.5 Strengths and limitations

This is the first Montenegrin dietary survey adhering to EFSA’s harmonized EU Menu methodology (17), enabling cross-country comparability and data integration. The standardized FoodEx2 classification and two 24-h recalls enhance validity, consistent with European methodology (16).

Nevertheless, self-reporting bias and underreporting, particularly among overweight individuals, remain inherent limitations of recall-based dietary assessments (42). This phenomenon is commonly discussed in the context of inverse causality or differential misreporting by body weight status. To explore this issue, an exploratory linear regression analysis was performed with reported daily energy intake as the dependent variable and body weight as the independent variable. The analysis revealed a statistically significant but weak association (*β* ≈ 15.6 kcal/kg; *R*^2^ = 0.15), indicating substantial inter-individual variability and suggesting potential underestimation of energy intake among participants with higher body weight. Given the cross-sectional nature of the data and the limitations of 24-h recalls, this analysis should be interpreted descriptively rather than as a precise quantification of underreporting bias.

To address the non-normal distribution of dietary intake variables, intake adequacy was evaluated using probability-based approaches derived from normalized intake distributions rather than direct comparisons to fixed cut-off values. Seasonal dietary variation could not be assessed due to data availability. Alcohol intake may also be underestimated, as alcoholic beverages are frequently underreported in 24-h recalls, particularly in populations where alcohol consumption is not consistently perceived as part of habitual dietary intake. Despite these limitations, the national representativeness of the sample and strict adherence to EFSA methodology provide a robust baseline for nutrition surveillance and evidence-based policy development in Montenegro.

Beyond its public health relevance, this study also contributes to regional and European data harmonization, strengthening Montenegro’s integration within the EFSA network and enabling cross- country comparisons of food consumption and nutrient exposure. The data generated provide a solid foundation for future research on dietary risks, food safety, and sustainability.

## 5 Conclusion

This study provides the first nationally representative assessment of energy and macronutrient intake among Montenegrin adults, conducted in accordance with the EFSA EU Menu harmonized methodology. The findings reveal a diet characterized by excessive fat and protein intake, insufficient carbohydrate contribution, and limited consumption of fruits and vegetables, indicating a pronounced food transition toward energy-dense and predominantly animal-based foods.

Improving the nutritional profile of the Montenegrin population will require coordinated efforts between policymakers, health professionals, educators, and the food sector. Sustained monitoring, nutrition education, and the development of evidence-based national dietary guidelines aligned with European standards are essential steps toward healthier and more sustainable diets in Montenegro.

## Data Availability

Publicly available datasets were analyzed in this study. This data can be found at: https://dcf.ec.europa.eu/index_en.

## References

[1] World Health Organization. WHO European regional obesity report 2022. Copenhagen: WHO Regional Office for Europe. (2022).

[2] Rippin HL Hutchinson J Evans CEL Jewell J Breda JJ Cade JE. National nutrition surveys in Europe: a review on the current status in the 53 countries of the WHO European region. Food Nutr Res. (2018) 62:1362. doi: 10.29219/fnr.v62.1362 PMC5917420 29720930

[3] European Food Safety Authority. Dietary reference values for nutrients summary report. Techn Report. (2017) 14. doi: 10.2903/sp.efsa.2017.e15121

[4] Vincze F Muka T Eichelmann F Llanaj E. Eating out intensity, ultra-processed foods and BMI among Albanian youth. Public Health Nutr. (2023) 26:2953–62. doi: 10.1017/S1368980023002173, 37842793 PMC10755451

[5] Gurinović M Milešević J Zeković M Knez M Takić M Šarac I Capacity development and harmonization of food consumption data collection in EFSA EU Menu National Dietary Surveys in Balkan Region-Building: the evidence base for diet monitoring and food systems transformation. In: The 14th European Nutrition Conference FENS (2023). Basel Switzerland: MDPI. 24.

[6] Monteiro CA Cannon G Levy RB Moubarac JC Louzada ML Rauber F . Ultra-processed foods: what they are and how to identify them. Public Health Nutr. (2019) 22:936–41. doi: 10.1017/S1368980018003762, 30744710 PMC10260459

[7] Monteiro CA Moubarac JC Levy RB Canella DS C Louzada ML Da Cannon G. Household availability of ultra-processed foods and obesity in nineteen European countries. Public Health Nutr (2018);21:18–26, doi: 10.1017/S1368980017001379, . 28714422 PMC10260838

[8] Spahija B Qirjako G Toci E Roshi E Burazeri G. Socioeconomic and lifestyle determinants of obesity in a transitional southeast European population. Med Arch. (2012) 66:16. doi: 10.5455/medarh.2012.66.s16-s20, 22937684

[9] Milešević J Zeković M Šarac I Knez M Krga I Takić M . Energy and macronutrient dietary intakes of Serbian adults 18–64 years old: EFSA EU menu food consumption survey in Serbia (2017–2022). Foods. (2025) 14:1228. doi: 10.3390/foods14071228, 40238476 PMC11988697

[10] Dokova KG Pancheva RZ Usheva NV Haralanova GA Nikolova SP Kostadinova TI . Nutrition transition in Europe: east-west dimensions in the last 30 years—a narrative review. Front Nutr. (2022) 9:919112. doi: 10.3389/fnut.2022.919112, 35873435 PMC9301044

[11] Gicevic S Gaskins AJ Fung TT Rosner B Sabanovic E Gurinovic M . Fueling an epidemic of non-communicable disease in the Balkans: a nutritional survey of Bosnian adults. Int J Public Health. (2019) 64:873–85. doi: 10.1007/s00038-019-01222-3, 30830244

[12] Gurinović M Nikolić M Zeković M Milešević J Kadvan A Ranić M . Implementation of harmonized food consumption data collection in the Balkan region according to the EFSA EU menu methodology standards. Front Nutr. (2022) 8:809328. doi: 10.3389/fnut.2021.809328 35127791 PMC8811292

[13] Šoher L Čačić Kenjerić D Pavlić M Rumbak I Šarlija N Ilić A . Macronutrient intake and food categories’ contribution to daily energy intake according to BMI in primary school children in Croatia. Nutrients. (2024) 16:4400. doi: 10.3390/nu16244400 39771021 PMC11679920

[14] Bjegovic V Kovacic L Laaser U. The challenge of public health transition in South Eastern Europe. J Public Health. (2006) 14:184–9. doi: 10.1007/s10389-006-0053-5

[15] Jasar D Curcic B. Common nutrition and health issues of food in the Balkans Nutritional and Health aspects of food in the Balkans Academic Press, London, United Kingdom (Elsevier). (2021). p. 279–97.

[16] van Rossum C ter Borg S Nawijn E Oliveira A Carvalho C Ocké M. Literature review on methodologies and tools for National Dietary Surveys; results of ERA EU-menu-project. EFSA Support Publ. (2022) 19:1–72. doi: 10.2903/sp.efsa.2022.EN-7725

[17] Martinovic A Labovic SB Orahovac A. National Dietary Survey on children in Montenegro from 1 to 9 Years old – External scientific Report. EFSA Support Publ. (2023) 20:1–31. doi: 10.2903/sp.efsa.2023.EN-8470

[18] Zekovic M Gurinovic M Milesevic J Kadvan A Glibetic M. National Food Consumption Survey among 10–74 Years old Individuals in Serbia. EFSA Support Publ. (2022) 19:1–30. doi: 10.2903/sp.efsa.2022.EN-7401

[19] Popovska S Jonovska K Adzija SS Mickova ST Spiroski I. National Dietary Survey on the children Population in the Republic of North Macedonia. EFSA Support Publ. (2022) 19:1–25. doi: 10.2903/sp.efsa.2022.EN-7169

[20] Sokolić D Kenjerić DČ Šoher L Colić-Barić I Rumbak I Ilić A . Croatian national food consumption survey on adolescents and adults from 10 to 99 years of age. EFSA Support Publ. (2024) 21:1–37. doi: 10.2903/sp.efsa.2024.EN-8577

[21] Milešević J Zeković M Šarac I Knez M Krga I Stevanović V Dietary patterns of Serbian adults 10–74 years old: Serbian National Food Consumption Survey following EU Menu methodology. In: The 14th European Nutrition Conference FENS 2023. Basel Switzerland: MDPI; (2024), 288.

[22] Gregori D French M Gallipoli S Lorenzoni G Ghidina M. Consumption of fruit and vegetables: the ROUND (world map of consumption of fruit and vegetables and nutrient deficits) project. Proc Nutr Soc. (2020) 79:E698. doi: 10.1017/S0029665120006473

[23] Kolaček S Hojsak I Radonić M Niseteo T Radunić A Marić L . Adherence to the Mediterranean diet in children and adolescents in the Mediterranean and continental regions of Croatia. Coll Antropol. (2022) 46:21–7. doi: 10.5671/ca.46.1.4

[24] Rippin HL Maximova K Loyola E Breda J Wickramasinghe K Ferreira-Borges C . Suboptimal intake of fruits and vegetables in nine selected countries of the World Health Organization European region. Prev Chronic Dis. (2023) 20:230159. doi: 10.5888/pcd20.230159, 37972606 PMC10684282

[25] Institut za javno zdravlje Crne Gore UG. Istraživanje o ishrani u Crnoj Gori 2022: Finalni izvještaj – jun 2023. Podgorica: Institute of Public Health of Montenegro. (2023).

[26] Gurinović M Milešević J Kadvan A Nikolić M Zeković M Djekić-Ivanković M . Development, features and application of DIET ASSESS and PLAN (DAP) software in supporting public health nutrition research in central eastern European countries (CEEC). Food Chem. (2018) 238:186–94. doi: 10.1016/j.foodchem.2016.09.114 28867092

[27] European Food Safety Authority (EFSA). EFSA Journal. (2014) 12:3944. 1–80. doi: 10.2903/j.efsa.2014.3944 PMC7163704 32313570

[28] European Food Safety Authority (EFSA). The food classification and description system FoodEx 2 (revision 2). EFSA Support Publ (2015) EN-804, 1–90. doi: 10.2903/sp.efsa.2015.EN-804

[29] Gurinovic M Milesevic J Zekovic M Kadvan A Ranic M Glibetic M. Capacity development in food and nutrition in central and Eastern Europe: a decade of achievements. Food Policy. (2020) 96:101850. doi: 10.1016/j.foodpol.2020.101850

[30] MacLean WC Warwick P. Food energy: methods of analysis and conversion factors: report of a technical workshop, Rome, 3–6 December 2002 Food and Agriculture Organization of the United Nations (2003). p. 87.

[31] El Bilali H Cardone G Bottalico F Ottomano Palmisano G Acquafredda A Capone R. Mediterranean diet in the Western Balkans. Agrofor. (2021) 6. doi: 10.7251/AGRENG2102077E

[32] Regional overview of food security and nutrition in Europe and Central Asia 2020 FAO, WFP, UN, UNICEF, WHO and WMO (2021) 1–140.

[33] Spiroski I Nikolić M Kochubovski M Gurinović M Ristovska G Kadvan A. Energy, macronutrients and dietary fibre intake among adults in North Macedonia. Cent Eur J Public Health. (2020) 28:24–32. doi: 10.21101/cejph.a5345, 32228813

[34] Rippin H Hutchinson J Jewell J Breda J Cade J. Adult nutrient intakes from current national dietary surveys of European populations. Nutrients. (2017) 9:1288. doi: 10.3390/nu9121288 29186935 PMC5748739

[35] Ruiz E Ávila J Valero T Del Pozo S Rodriguez P Aranceta-Bartrina J . Energy intake, profile, and dietary sources in the Spanish population: findings of the ANIBES study. Nutrients. (2015) 7:4739–62. doi: 10.3390/nu7064739, 26076230 PMC4488811

[36] Martimianaki G Peppa E Valanou E Papatesta EM Klinaki E Trichopoulou A. Today’s Mediterranean diet in Greece: findings from the National Health and nutrition survey—HYDRIA (2013–2014). Nutrients. (2022) 14:1193. doi: 10.3390/nu14061193, 35334847 PMC8949101

[37] French Agency for Food E and OH and S (ANSES). INCA 3: changes in consumption habits and patterns — results of the French individual and National Study on Food Consumption (2014–2015). Maisons-Alfort, France: (2017).

[38] Bennett E Peters SAE Woodward M. Sex differences in macronutrient intake and adherence to dietary recommendations: findings from the UK biobank. BMJ Open. (2018) 8:e020017. doi: 10.1136/bmjopen-2017-020017, 29691247 PMC5922487

[39] Bulotaitė G Bartkevičiūtė R Barzda A Stukas R. Intakes of energy, macronutrients, and micronutrients in adult Lithuanian population: a national study of 2019–2020. J Nutr Sci. (2024) 13:e46. doi: 10.1017/jns.2024.40, 39469191 PMC11513885

[40] Lombardo M Feraco A Armani A Camajani E Gorini S Strollo R . Gender differences in body composition, dietary patterns, and physical activity: insights from a cross-sectional study. Front Nutr. (2024) 11:1414217. doi: 10.3389/fnut.2024.1414217, 39055386 PMC11271261

[41] Gicevic S Gaskins AJ Fung TT Rosner B Sabanovic E Milesevic J . Demographic and socio-economic predictors of diet quality among adults in Bosnia and Herzegovina. Public Health Nutr. (2019) 22:3107–17. doi: 10.1017/S1368980019001988, 31397250 PMC10260543

[42] Okada E Nakade M Hanzawa F Murakami K Matsumoto M Sasaki S . National nutrition surveys applying dietary records or 24-h dietary recalls with questionnaires: a scoping review. Nutrients. (2023) 15:4739. doi: 10.3390/nu15224739, 38004132 PMC10674720

